# High-Throughput Phototactic Ecotoxicity Biotests with Nauplii of *Artemia franciscana*

**DOI:** 10.3390/toxics10090508

**Published:** 2022-08-29

**Authors:** Yutao Bai, Jason Henry, Tomasz M. Karpiński, Donald Wlodkowic

**Affiliations:** 1 The Neurotox Lab, School of Science, RMIT University, Plenty Road, P.O. Box 71, Bundoora, VIC 3083, Australia; 2 Chair and Department of Medical Microbiology, Poznań University of Medical Sciences, Wieniawskiego 3, 61-712 Poznań, Poland

**Keywords:** ecotoxicology, behavior, phototaxis, *Artemia franciscana*, brine shrimp

## Abstract

Analysis of sensorimotor behavioral responses to stimuli such as light can provide an enhanced relevance during rapid prioritisation of chemical risk. Due to technical limitations, there have been, however, only minimal studies on using invertebrate phototactic behaviors in aquatic ecotoxicity testing. In this work, we demonstrate an innovative, purpose-built analytical system for a high-throughput phototactic biotest with nauplii of euryhaline brine shrimp *Artemia franciscana*. We also, for the first time, present a novel and dedicated bioinformatic approach that facilitates high-throughput analysis of phototactic behaviors at scale with great fidelity. The nauplii exhibited consistent light-seeking behaviors upon extinguishing a brief programmable light stimulus (5500K, 400 lux) without habituation. A proof-of-concept validation involving the short-term exposure of eggs (24 h) and instar I larval stages (6 h) to sub-lethal concentrations of insecticides organophosphate chlorpyrifos (10 µg/L) and neonicotinoid imidacloprid (50 µg/L) showed perturbation in light seeking behaviors in the absence of or minimal alteration in general mobility. Our preliminary data further support the notion that phototactic bioassays can represent an attractive new avenue in behavioral ecotoxicology because of their potential sensitivity, responsiveness, and low cost.

## 1. Introduction

There is a growing body of evidence suggesting both acute and chronic exposure to low concentrations of diverse chemicals can lead to pleiotropic perturbations in aquatic animal behaviors [1,2,3,4,5,6,7,8]. From the perspective of ecotoxicology and chemical risk assessment, behavioral endpoints are widely considered sensitive and highly integrative phenotypic biomarkers of exposure to sub-lethal concentrations of industrial pollutants [9,10,11].

The increasing number of pollutants with the potential to modify behavior is poised to necessitate a paradigm shift from labor-intensive, low-throughput behavioral tests to alternatives with higher throughput [11,12,13,14]. The latter are commonly addressed by applying swimming speed alteration tests with small aquatic invertebrates [15,16]. Such tests have become particularly popular due to their low costs and minimal infrastructure requirements, as well as sensitivity for measuring the impact of behavior-modifying compounds [17,18].

There have been several excellent reports demonstrating examples of automated assays utilizing spontaneous swimming speed alteration in a microperfusion environment, as well as a static, multi-well plate system with larval stages of brine shrimp (*Artemia franciscana*), amphipods, neonates of water fleas (*Daphnia magna*) and even larval stages of zebrafish (*Danio rerio*) [19,20,21,22,23]. However, so far, there has been only a handful of studies exploring invertebrate phototactic behaviors at higher throughput [22,24,25]. Those predominantly used commercial animal tracking systems designed for analyzing zebrafish and did not enable straightforward customization in terms of tests chamber size and geometry, as well as the fine-tuning of the photic stimulus, including its size and shape [12,13].

Phototaxis is characterized by directional movement towards (positive phototaxis) or away from (negative phototaxis) from an illuminated area, respectively [22,26,27,28]. The phototactic responses depend on the levels of neuronal complexity. The simplest form of phototaxis is non-visual scanning phototaxis known in planktonic dispersing larvae across multiple phyla [27]. It is activated in response to the sensory input from the low-resolution visual eyes containing two cells, a sensory-motor photoreceptor and a shading pigment cell [27,28]. The sensory-motor photoreceptor directly innervates the effector’s muscles. Since only one photoreceptor cell is present, the animal relies on body rotation or other scanning movements to detect the direction of the light [27].

More complex eye receptors are responsible for visual phototaxis, also known as spatial vision. Visual phototaxis can sense the direction and intensity differences in environmental light levels without scanning movements since it relies on at least two photoreceptors pointing in different directions [27]. It usually requires two or more cerebral photoreceptor cells [27]. Bilateral neural circuit divergence and integration of a motor neuron into the photo-sensing circuit are required for switching between positive and negative phototaxis activated by the same eye [27,29]. Further evolution of photoreceptors affords low-resolution image-forming eyes [27,28,30]. During visual phototaxis, the photic inputs can be integrated with an array of other sensory inputs by the central nervous system before regulating the phototactic turns [27,28].

Phototactic behaviors are critically important for the zooplankton invertebrates and contribute to their vertical migration patterns and facilitate larval dispersal [27,28,29]. Indeed, many zooplankton species, including brine shrimp, exhibit nocturnal diel vertical migration (DVM), ascending near the surface during low light and descending to deeper, dimly lit areas to avoid predators during the day [22]. Older larval stages are known to be negatively phototactic. This enables them to migrate toward the benthic zone before larval settlement [27]. Many larval stages of zooplankton can switch between positive and negative phototaxis, enabling adaptations to preferred water depth, food availability, and the presence of predators [27]. Negative phototaxis is also important to avoid irradiation with ultraviolet light (UV) [31]. It has been demonstrated, for instance, that strong photoirradiation of planktonic sea urchin larvae immediately drives them away from the surface due to backward swimming [31]. Environmental factors such as light intensity and its spectral distribution, temperature, oxygen content, salinity, and chemical pollutants can modify the strength of phototaxis [27]. Indeed, the recently published reports provided pilot evidence that applications of phototactic responses in invertebrates can provide new protocols for ecotoxicity testing [11,22,23,24,32,33,34].

In this work, we demonstrate for the first time a purpose-built and low-cost system for high-throughput light-seeking biotests with nauplii of the euryhaline crustacean *Artemia franciscana*, widely used in a swimming speed alteration (SSA) assay developed by Faimali et al. [17,18]. We also present a novel and dedicated bioinformatic approach that facilitates high-throughput analysis of invertebrate phototactic behaviors with great fidelity. *Artemia* nauplii immobilization, as well as SSA tests, are widely used in marine ecotoxicity worldwide because of its easy rearing, short life cycle, large offspring production as well as easy manipulation [16,17,18,35,36]. The popularity and broad use of this model persist despite the ongoing discussion about its validity in marine toxicity testing because of the absence of this genus from marine ecosystems and the reported lack of robust sensitivity to many pollutants [35,36].

Overall, we showcase the added value of phototactic bioassays for preliminary screening of emerging contaminants in aquatic ecotoxicology.

## 2. Materials and Methods

### 2.1. Chemicals and Test Organisms

Organophosphate (OP) insecticide chlorpyrifos (Dr. Ehrenstorfer GmbH, Augsburg, Germany, purity 99.5%, 10 µg/L final concentration) and neonicotinoid insecticide imidacloprid (Sapphire Bioscience Pty Ltd., Redfern, NSW, Australia, purity >98%, 50 µg/L final concentration) were purchased as solid powders. Stock solutions were prepared by dissolving the compounds in dimethylsulfoxide (DMSO, Sigma-Aldrich, Melbourne, VIC, Australia) each day before experiments and stored in the fridge at 4 °C. All test concentrations were made up of serial dilutions of the stock solution in filtered seawater (RMIT Aquatic Facility, Bundoora, VIC, Australia). Maximum concentrations of DMSO used in all experiments did not exceed 1 × 10^−5^% (*v*/*v*).

Dehydrated eggs of the marine crustacean *Artemia franciscana* (Southern Biological Pty Ltd., Melbourne, VIC, Australia) were used as before [19,23]. All chemical exposures were conducted by immersing approximately 200 eggs in an 85 mm Petri dish filled with 35 mL of filtered seawater (pH 8.0 ± 0.5) spiked with tested chemical solutions or vehicle controls [19,23]. Samples were then incubated at a constant stabilized temperature (22 °C) under illumination of 3500 ± 500 lux. After 24 h of egg hydration, five hatched nauplii at the stage of nauplius I were randomly selected and transferred to each well of the test plates. Each well was filled with 2 mL of identical chemical solution such as the one used during embryo development. The behavioral analysis was performed at the 6 h post-hatching (hph). Each exposure was performed in at least 5 independent replicates, each comprising 240 animals.

Before any experiments, animals were allowed to acclimate on test plates under dark conditions illuminated only by infrared (IR) light for 20 min. The analysis consisted of a 5 min pre-stimulus dark phase, a 20 min light stimulus phase, and a 20 min dark post-stimulus recovery phase. Animal behavior was continuously video recorded in each of the phases.

### 2.2. Custom High-Throughput Behavioral Analysis System

**Figure 1:**
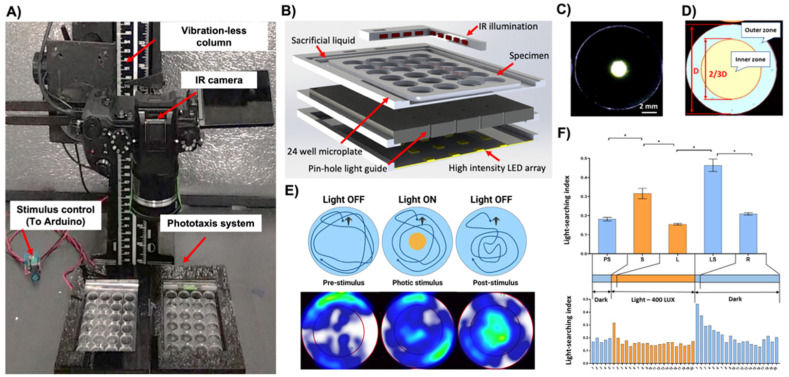
Design and validation of the high-throughput system for the analysis of phototactic behaviors of *Artemia franciscana*. (**A**) A high-definition IR camera mounted on the vibration-less column to enable imaging of two 24-chamber custom test plates simultaneously; (**B**) Multi-layer construction of the phototaxis test plate with (i) orthogonal IR illumination, (ii) test chambers, (iii) pinhole light guide layer, (iv) LED light stimulus array; (**C**) The photic stimulus providing a narrow beam of light with only a discrete illuminated area at the epicenter of the chamber; (**D**) Bioinformatic analysis in animal tracking software to account for uniform volume and area size of the inner/outer regions and the relative occupancy of animals; (**E**) A cartoon and occupancy heatmaps depicting the behavioral responses including the light searching behaviors (LSBs) upon extinguishing of the photic stimulus; (**F**) Discrete aggregate behavioral fingerprints (top) with a corresponding and expanded time-resolved analysis (bottom). PS—pre-stimulus (light OFF), S—startle (light ON), L—adaptation to light (Light ON), LS—light searching behaviors (light OFF), R—recovery (light OFF). At least four independent experiments were performed, averaging data from up to 240 specimens. * *p* < 0.05 one-way ANOVA.

The system consisted of seven modules: (i) the high-definition camera Panasonic Lumix G7 digital camera (Panasonic Australia Pty Ltd., Macquarie Park, NSW, Australia) converted to allow the sensor to receive infrared light (IR), equipped with a 30 mm macro-objective lens (Olympus, Tokyo, Japan) with a high pass IR filter (>850 nm) and mounted on a vibration-less photographic column (Polaroid M3, Polaroid Inc., Minnetonka, MN, USA) inside an isolation chamber; (ii) a transparent 24-chamber custom test plate; (iii) photic stimulus LED panel with associated electrical wiring, (iv) pinhole layer, (v) an orthogonal infrared (IR) illumination system; (vi) a video data stream capture PC computer equipped with a High-Definition Multimedia Interface (HDMI) interface card (BlackMagic 4K Decklink Mini card, BlackMagic Design, Port Melbourne, VIC, Australia) and an open-source video recording software (Open Broadcaster Software Studio, https://obsproject.com/ accessed on 2 August 2022); (vii) a photic stimuli control software (Figure 1A,B).

The disposable custom test plates (125 × 75 × 4 mm, L × W × H) featured 24 wells (15.6 × 3 mm, ø × H) with a nominal volume of 574 µL. The test chambers were surrounded by an engraved channel filled with a sacrificial liquid (10 mL) to reduce the evaporative loss of the medium from the test chambers during the long-term experiment (Figure 1B). Test plates were designed using a SolidWorks 2016 (Dassault Systems SolidWorks Corp, Concord, MA, USA) software and fabricated in a biocompatible poly(methyl methacrylate) (PMMA) thermoplastic using a 30W infrared laser micromachining system (Universal Laser Systems, Scottsdale, AZ, USA) as described before [37,38].

The LED light stimulus panel (400 Lux per LED, 5500K) was custom built so that the position of each LED was concentric with the center of the respective test chamber (Figure 1C–E). The pinhole layer was sandwiched between the test plate and LED panel, providing a narrow beam of light. It effectively created an illuminated circular area at the center of the chamber (ø 2 mm) (Figure 1C). Since the camera lens was equipped with a high pass IR filter, the photic stimulus was not visible via the sensor, thus not affecting the exposure of the video files. The orthogonal infrared (IR) illumination provided a uniform, shadow-free illumination of the entire test plate.

The operation of the photic stimulus LED panel was programmable using an in-house developed software interface written in C# using Microsoft Visual Studio 2017 (Microsoft, Redmond, WA, USA). It enabled the setup of trial parameters, such as light stimuli duration and the number of ON/OFF cycles, to create any light sequence for an independent or time-lapse experiment (Supplementary Figure S1A). The graphical user interface (GUI) also provided trial events and temperature logging to monitor experimental conditions inside the closed video acquisition chamber.

### 2.3. High-Throughput Bioinformatic Analysis

The high-definition recording allowed for up to two 24-well custom plates with a total of 240 organisms (48 chambers with 5 individuals per well) to be captured simultaneously while preserving sufficient resolution to identify and track all organisms. Acquired video files were post-processed using a high-throughput batch processing protocol described earlier [5,23].

Briefly, a digital background mask image in which each test chamber arena was filled in white color was created using Adobe Photoshop CC 2017 (Adobe, San Jose, CA, USA). This mask was imported with respective video files into a DaVinci Resolve 16 video editing software (Blackmagic Design, Port Melbourne, VIC, Australia). This allowed for digital background subtraction and artifact-free optimized video files in which each arena has a completely white background area, while the organisms are represented as clearly distinguishable dark objects.

High-throughput animal tracking on optimized video files was performed using Ethovision XT ver. 16 (Noldus Information Technology, Wageningen, The Netherlands) as described earlier [5,23]. To analyze many samples at a considerable throughput, a combination of multi-arena and social interaction modules in Ethovision software provided us with a unique capability to simultaneously all track animals in 48 arenas (a total of 240 animals) with a detection rate >90%. Automatic frame-by-frame tracking produced time-stamped x,y coordinate pairs assigned to centroids of detected objects and provided a foundation for the reconstruction of graphical animal trajectories and behavioral parameters (i.e., average distance traveled, time spent in arena zone) calculated for each test subject. Raw numerical data sets were exported as Excel files.

### 2.4. Statistical Analysis

Statistical analysis was performed using IBM SPSS Statistics, version 26 (IBM Corp., Armonk, NY, USA) and GraphPad Prism 8 software suite (Graph Pad, San Diego, CA, USA). The data averaged from one-minute time bins was used for analysis as described in detail before [23].

To quantify the phototactic responses, a light searching index (LSI) was used and defined by dividing the time spent in the inner zone of the well by the total time of the arena as the inverse of the wall preference index (Tw) described before (Figure 1D,E) [23]. To investigate the effect of behavioral conditions upon the organism’s phototactic response, the LSI was partitioned into five distinct fingerprints. For each phase, the averaged LSI from all time bins was compared with its previous phase using a one-way analysis of variance (ANOVA) followed by a Turkey test.

To investigate the effect of behavioral conditions and toxicant exposure upon the organism’s phototactic response, the LSI from each phase of control and treatment groups were analyzed using a two-way ANOVA, followed by Bonferroni corrections to account for the multiple comparisons.

To investigate the effect of behavioral conditions and toxicant exposure upon the organism’s swimming speed alteration, the total moving distance from each light condition of control and treatment groups was analyzed using a two-way ANOVA, then Bonferroni corrections were applied to account for the multiple comparisons.

Data presented in charts were expressed as means ± standard error (SE) of the replicates. All data sets were checked for normality of distribution using Shapiro-Wilk’s test prior to hypothesis testing, and data sets were considered significantly different when *p* < 0.05.

## 3. Results

The pinhole design of the device permitted discrete and focused illumination of a small circular area at the center of each chamber. The light diffusion gradient around the edge of the central zone was limited to approximately 1 mm (Figure 1C). This effectively created two distinct areas in each chamber: (i) a circumference dark and (ii) a central light zone (Figure 1C).

To account for uniform volume and area size and thus the relative occupancy of animals in the inner and outer zones, their geometry parameters were defined according to the description of the Tw index [23,39]. The inner zone was defined as a 2/3 diameter of the entire test well in the animal tracking software (Figure 1D,E). In real-world dimensions, this translated to a diameter of 10.4 mm. The inner and outer zones thus comprised approximately 45% and 55% of the nominal chamber volume, respectively.

In the darkness, the nauplii exhibited strong wall preference behaviors (LSI = 0.18 ± 0.0.01; Mean ± SE; Figure 1E,F). Upon activation of the 400-lux photic stimulus, they showed a phototactic behavior and migrated towards the central region of the chamber (positive phototaxis; LSI 0.32 ± 0.03; Mean ± SE; *p* < 0.001; Figure 1E,F). The time-resolved analysis revealed that this startle response was transient and lasted for about 1 min. Afterward, the animals returned to behavioral characteristics of the pre-stimulus phase (LSI 0.16 ± 0.004; Mean ± SE, *p* < 0.001; Figure 1F). Upon extinguishing of the photic stimulus, the nauplii displayed very strong light searching behaviors (LSBs) and migrated vigorously to the central region (LSI 0.46 ± 0.03; Mean ± SE; *p* < 0.001; Figure 1E,F). The time-resolved analysis indicated that LSBs and exploration of the extinguished light zone were strongest for approximately 1 min. Subsequently, animals recovered to their native dark phase behaviors with a continuously diminishing intensity of LSBs during, on average, 15 min (LSI 0.21 ± 0.01; Mean ± S; *p* < 0.001; Figure 1F).

The intensity and duration of LSBs were directly proportional to the intensity of the light and plateaued at approximately 400–800 lux (*p* < 0.001 (Supplementary Figure S1B). For the subsequent chemical tests, the light startle and LSBs endpoints were defined as responses during the first minute upon turning on and off the photic stimulus (Figure 1F).

**Figure 2:**
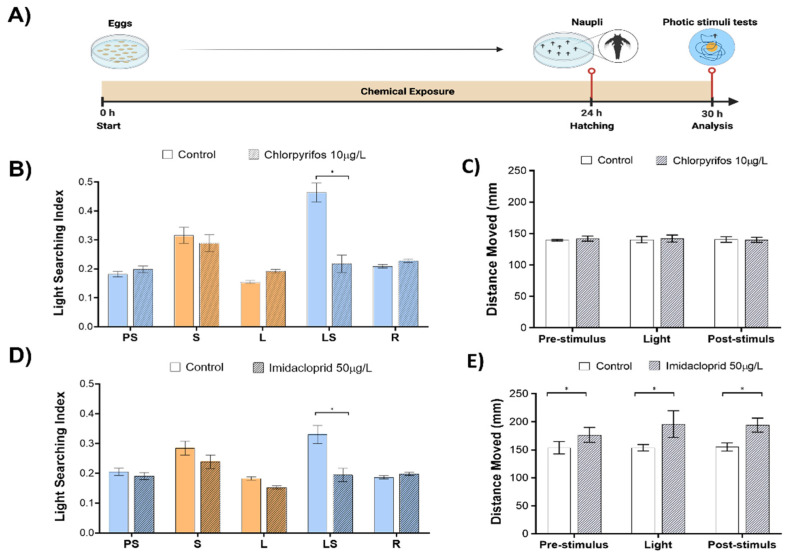
Phototactic responses of larval brine shrimp exposed to sub-lethal concentrations of insecticides. (**A**) experiment workflow depicting the phototactic test protocol; (**B**) phototactic fingerprints upon exposure to organophosphate chlorpyrifos (10 µg/L); (**C**) animal overall motility (total distance moved in mm) analyzed in each phase of light OFF-ON-OFF cycle upon exposure to chlorpyrifos (10 µg/L); (**D**) phototactic fingerprints upon exposure to neonicotinoid imidacloprid (50 µg/L); (**E**) animal overall motility (total distance moved in mm) analyzed in each phase of light OFF-ON-OFF cycle upon exposure to imidacloprid (50 µg/L). At least four independent experiments were performed, averaging data from up to 240 specimens. * *p* < 0.05 one-way ANOVA. Figure created using www.BioRender.com accessed on 2 August 2022.

Next, we set to explore in a proof-of-concept validation if sub-lethal effects of insecticide pollutants such as organophosphate chlorpyrifos (10 µg/L) and neonicotinoid imidacloprid (50 µg/L) will induce perturbations of phototactic behaviors and how such sensory motor endpoints compare with the general motility readout (Figure 2A). The concentrations of both insecticides did not induce any delay in the hatching of eggs or mortality during the exposure.

The exposure to chlorpyrifos induced a significant reduction of LSBs (53.1% change to control, *p* < 0.001, Figure 2B) in the absence of any observable effects in the pre-stimulus, light, and recovery phases. Animal motility, represented as the total distance moved in each of the analyzed phases, was unaffected by the chlorpyrifos (Figure 2C).

The exposure to imidacloprid induced a significant reduction of LSBs (41.1% change to control, *p* < 0.001; Figure 2D) without any observable effects in the pre-stimulus, light, and recovery phases. Animal motility was increased by 13 % (*p* = 0.03), 21.6 % (*p* < 0.001), 20.1% (*p* < 0.001) in pre-stimulus, light and recovery phases, respectively (Figure 2E).

## 4. Discussion

The prospects and unique value of utilizing invertebrate phototactic behaviors in aquatic ecotoxicology were postulated by Sounders et al. and Delupis and Rotondo in 1988 [26,40]. From a perspective of eco-neurotoxicology, the implementation of sensory-motor assays over simple non-stimulated swimming biotests can provide a deeper insight into the impact of environmental toxicants on central nervous systems [7,11]. Interestingly, although many invertebrates reportedly exhibit phototactic behaviors, there is a notable paucity in the characterization and exploration of such sensorimotor assays in behavioral ecotoxicology [11]. Since the early seminal work, there have been a handful of demonstrations of phototaxis in toxicity tests utilizing predominantly *Daphnia magna*, marine and freshwater amphipods (*Echinogammarus marinus* and *Gammarus pulex*), and brine shrimp [22,24,25,41,42,43]. An emerging application of higher throughput phototaxis tests has been demonstrated by Simao et al. and Bedrossiantz et al. for photomotor response assay (DPRA) that measures the vertical negative phototaxis of daphnids after a sudden increase in light intensity [44,45]. However, these studies analyzed the distance moved after a sudden increase in light intensity rather than actual phototactic responses defined as a migration of the animals towards or away from the light stimulus.

The lack of user-friendly, cost-effective, and easily customizable analytical technologies facilitating straightforward phototactic biotests is one of the reasons behind the lack of data on this emerging topic [11,12,33]. To address this, we have developed a low-cost, open-source system capable of analyzing up to 48 phototactic test chambers for small aquatic model organisms such as nauplii of *Artemia franciscana*. The cost of the system, including the camera, is <1000 USD. Although the additional cost of the commercial animal tracking software such as the Ethovison XT is substantial, the analysis can also be performed, albeit at a lower throughput, with many open-source and thus free animal tracking software such as, e.g., ToxTrac [12,23].

Although we selected the configuration with a central light stimulus, the fabrication of plates with a commercial laser cutter allows for considerable design flexibility and modification in terms of depth, geometry, as well as size and placement of the photic zone [23]. An innovative orthogonal infrared illumination system provided a uniform and artifact-free illumination of the test plates at the 90-degree angle. This spatially separates the IR and light stimulus to enable the incorporation of diverse shapes and configurations of the photic stimuli. In contrast, all existing commercial behavioral analysis systems employ IR illumination in the same plane as the photic stimulus (bottom illumination principle), preventing the creation of custom-defined, discrete light and dark zones [21,22].

In the absence of light stimulus, freshly hatched nauplii (first instar developmental stage) of *Artemia franciscana* exhibited a strong innate wall preference (wall hugging) behavior consistent with earlier reports [22,23]. The activation of the defined light zone induced rapid phototactic behaviors characterized by a transient migration of the specimens towards the illuminated central zone. Upon extinguishing the light stimulus, the nauplii also displayed very strong and consistent light searching behaviors (LSBs). A recent excellent study by Kohler et al. exploited a commercial behavioral analytical system with relatively large areas of illumination for the global analysis of *Artemia* sp. phototaxis [22]. In contrast, the design of our device utilized a small and tightly controlled light zone stimulus with minimal light bleeding. To the best of our knowledge, this is the first demonstration that the early larval stages of brine shrimp show prolonged light searching behaviors upon extinguishing the light source. The historic report by Delupis and Rotondo indicated that depending on the intensity of the light stimulus, *Artemia* may show a switch between positive and negative phototaxis [26]. It has also been postulated that the transition from light to dark phase may trigger startle behaviors, increase exploratory behavior, or increase searching for light areas [22]. Many zooplankton species, including brine shrimp, exhibit nocturnal diel vertical migration (DVM), ascending to near the surface during low light and descending to deeper, dimly lit areas to avoid predators during the day [22].

Our preliminary ecotoxicity data show that sub-lethal concentrations of organophosphate chlorpyrifos and neonicotinoid imidacloprid induced strong perturbations in LSBs fingerprint. Both insecticides were chosen in our study for the preliminary validation because they specifically affect the central nervous system. They are not intended for aquatic use, but their residues are frequently detected in surface waters that drain agricultural lands [46,47]. They can subsequently enter the estuarine environments depending on the local conditions. Median neonicotinoid surface water concentrations are on the order of ng/L, but peak concentrations can achieve low μg/L levels. The environmentally relevant concentrations of organophosphates are reportedly in the range of 0.1–2.55 μg/L [48]. There is also a significant range of susceptibility to neonicotinoids and organophosphates among reported zooplankton crustacean species [46]. For instance, *D. magna* has been reported to be one of the most sensitive species in short-term (48 h EC50 = 56.6 mg/L) and long-term (21 d NOEC = 1.25 mg/L) exposure to imidacloprid [49]. Importantly there is a paucity of quantitative studies on the effects of both insecticides on the sensory-motor behavior of aquatic invertebrates. The concentrations employed in our study exceed the environmentally relevant levels, but they were only intended as a proof-of-concept to demonstrate the new technology in combination with potential perturbations in the sensory-motor behavioral responses.

In the case of chlorpyrifos, the sensory-motor perturbations were discrete since, at the chosen nominal concentration, the insecticide did not induce any change in overall locomotory activity. The latter endpoint is used, for instance, in the swimming speed alteration assay developed by Faimali et al. [16,18]. This indicates that phototactic behaviors might be used to develop more sensitive indices. Of note, we have modified the protocol of the conventional SSA because we aimed to highlight the development of an accelerated biotest. Accordingly, our regimen included exposure to chemicals already during hydration and development of the eggs for 24 h, followed by the short-term exposure of instar I stage for just 6 h. Conventional SSA and toxicity biotests commonly expose the nauplii at 30 h post-hatching when they are at the instar II–III stage. As suggested by some studies, the thick envelope of cysts can limit the penetration of chemicals into brine shrimp embryos [48]. A recent study by Gambardella et al. showed that exposure to low concentrations of OPs during brine shrimp embryo development could delay its development without notable developmental abnormalities [48]. Accordingly, we have employed a protocol with additional 6-h exposure of the first instar stages as reported by us previously [19,23].

We acknowledge that our findings are only a proof-of-concept validation of the new open-source phototactic analysis system. Nevertheless, we postulate that wider exploration of invertebrate phototactic behaviors warrants further exploration in aquatic toxicity testing especially considering recent reports that support this notion [22,23,26,42,44,45,50]. However, we also recognize that *Artemia* sp. nauplii, although commonly used in marine ecotoxicity biotests, are known to be resistant to many chemicals [13,19,35,36,51,52]. Since the presented technology allows for easy operation and rapid readout, it will be tremendously interesting to prospectively explore phototactic behaviors in diverse aquatic invertebrates exposed to a range of behavior-modifying pollutants at environmentally relevant concentrations in diverse exposure regimens (e.g., acute, chronic, and multi-generational).

## 5. Conclusions

Our results demonstrate the utility of purpose-built analytical technology in exploring light-dark preference in small aquatic invertebrates. Given the short duration and repeatability of rapid phototactic responses, they warrant further exploration as endpoints in rapid prioritization pipelines for selecting pollutants with neurotoxic and neuromodulating properties for more advanced behavioral tests. We envisage that the presented technology, with its analytical scope and potential for further development, offers new and exciting avenues for exploring diverse combinations of abiotic, biotic (e.g., parasitic infections), and chemical stressors on aquatic invertebrate models.

## Data Availability

Sample video datasets generated and/or analyzed during the current study are available online from the author on request.

## Supplementary Materials

The following supporting information can be downloaded at: https://www.mdpi.com/article/10.3390/toxics10090508/s1, Figure S1: (A) A graphical user interface (GUI) of the in-house developed software to provide a programmable control of the photic stimulus. The software was written in C# using Microsoft Visual Studio 2017 (Microsoft, USA). It supports set up of trial parameters, such as duration of the light stimuli and the number of ON/OFF cycles. This enables creation of any light sequence for independent or time-lapse experiments, (B) The intensity and duration of light searching behaviors (LSBs) is directly proportional to the intensity of the light and plateaus at approximately 400-800 lux. LSI—light searching index; * *p* < 0.001.
