# Supplementary material for: High-Throughput Phototactic Ecotoxicity Biotests with Nauplii of Artemia franciscana
Source: Toxics. 2022 Aug 29;10(9):508. doi: 10.3390/toxics10090508 (PMC9501151; doi:10.3390/toxics10090508)
Supplement: Supplementary file 1 [file toxics-10-00508-s001.zip › toxics-1874602-supplementary.pdf]

toxics

MDPI

Supplementary Materials: High-Throughput Phototactic Ecotoxicity Biotests with Nauplii of *Artemia franciscana*

Yutao Bai, Jason Henry, Tomasz M. Karpiński, and Donald Wlodkowic

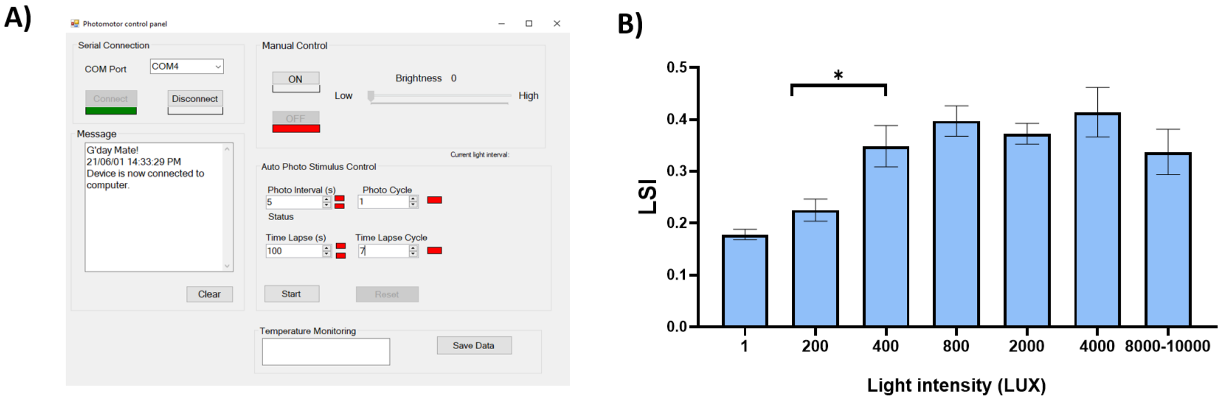

Figure S1. (A) A graphical user interface (GUI) of the in-house developed software to provide a programmable control of the photic stimulus. The software was written in C# using Microsoft Visual Studio 2017 (Microsoft, USA). It supports set up of trial parameters, such as duration of the light stimuli and the number of ON/OFF cycles. This enables creation of any light sequence for independent or time-lapse experiments, (B) The intensity and duration of light searching behaviors (LSBs) is directly proportional to the intensity of the light and plateaus at approximately 400–800 lux. LSI – light searching index; * $P < 0.001$.
